# Deep learning predicts immune checkpoint inhibitor-related pneumonitis from pretreatment computed tomography images

**DOI:** 10.3389/fphys.2022.978222

**Published:** 2022-07-25

**Authors:** Peixin Tan, Wei Huang, Lingling Wang, Guanhua Deng, Ye Yuan, Shili Qiu, Dong Ni, Shasha Du, Jun Cheng

**Affiliations:** ^1^ Department of Radiation Oncology, Guangdong Provincial People’s Hospital, Guangdong Academy of Medical Sciences, Guangzhou, China; ^2^ National-Regional Key Technology Engineering Laboratory for Medical Ultrasound, Guangdong Key Laboratory for Biomedical Measurements and Ultrasound Imaging, School of Biomedical Engineering, Health Science Center, Shenzhen University, Shenzhen, China; ^3^ Medical Ultrasound Image Computing (MUSIC) Laboratory, Shenzhen University, Shenzhen, China; ^4^ Marshall Laboratory of Biomedical Engineering, Shenzhen University, Shenzhen, China; ^5^ Department of Oncology, Guangdong Sanjiu Brain Hospital, Guangzhou, China

**Keywords:** immune checkpoint inhibitor-related pneumonitis, deep learning, transfer learning, contrastive learning, CT images, lung cancer

## Abstract

Immune checkpoint inhibitors (ICIs) have revolutionized the treatment of lung cancer, including both non-small cell lung cancer and small cell lung cancer. Despite the promising results of immunotherapies, ICI-related pneumonitis (ICIP) is a potentially fatal adverse event. Therefore, early detection of patients at risk for developing ICIP before the initiation of immunotherapy is critical for alleviating future complications with early interventions and improving treatment outcomes. In this study, we present the first reported work that explores the potential of deep learning to predict patients who are at risk for developing ICIP. To this end, we collected the pretreatment baseline CT images and clinical information of 24 patients who developed ICIP after immunotherapy and 24 control patients who did not. A multimodal deep learning model was constructed based on 3D CT images and clinical data. To enhance performance, we employed two-stage transfer learning by pre-training the model sequentially on a large natural image dataset and a large CT image dataset, as well as transfer learning. Extensive experiments were conducted to verify the effectiveness of the key components used in our method. Using five-fold cross-validation, our method accurately distinguished ICIP patients from non-ICIP patients, with area under the receiver operating characteristic curve of 0.918 and accuracy of 0.920. This study demonstrates the promising potential of deep learning to identify patients at risk for developing ICIP. The proposed deep learning model enables efficient risk stratification, close monitoring, and prompt management of ICIP, ultimately leading to better treatment outcomes.

## 1 Introduction

Since the first immune checkpoint inhibitor (ICI) ipilimumab was approved by the Food and Drug Administration for treating melanoma in 2011, ICIs have become standard treatments for many cancers such as lung cancer, renal cell carcinoma, Hodgkin lymphoma, and hepatocellular carcinoma (Akinleye and Rasool, 2019; Xin Yu et al., 2019; Robert, 2020; Vaddepally et al., 2020). Although ICIs produce remarkable immune response by immune upregulation and demonstrate improved cancer-related outcomes, they induce a unique spectrum of toxicities, called immune-related adverse events (irAEs) (Martins et al., 2019; Ramos-Casals et al., 2020). These irAEs can occur in multiple organ systems, where uncontrolled immune response is generated against healthy tissue. Due to different organ systems affected, there are various types of irAEs, including dermatitis, encephalitis, uveitis, hepatitis, and pneumonitis. Among these, immune checkpoint inhibitor-related pneumonitis (ICIP) is one of the most concerned adverse events because it is potentially life-threatening (Naidoo et al., 2017).

Lung cancer is the second most common cancer and the leading cause of cancer death worldwide. ICIs have shown significant clinical benefit in the treatment of advanced non-small cell lung cancer (NSCLC) (Reck et al., 2016). The incidence of ICIP in NSCLC is 4.1% as reported in a prospective study, while some real-word studies outside of clinical trials report a much higher incidence, ranging from 7% to 19% (Sears et al., 2019; Atchley et al., 2021). The time to onset of ICIP can vary from 9 days to 24.3 months after the initiation of immunotherapy (Nishino et al., 2015; Naidoo et al., 2017), with a median time of 52.5 days (Atchley et al., 2021). Most patients with ICIP have high severity that requires hospitalization, and about 27% of them die during the treatment for ICIP (Atchley et al., 2021). Unfortunately, the pathogenesis of ICIP has not been clearly elucidated. Possible risk factors include prior thoracic radiotherapy, pulmonary comorbidities, smoking status, and PD-1 inhibitors (Howell et al., 2015; Delaunay et al., 2017; Khunger et al., 2017; Pillai et al., 2018; Winer et al., 2018). However, it is challenging to accurately predict ICIP based on these clinical risk factors. In order to improve lung cancer treatment and outcomes, there is an urgent need for early prediction of ICIP, which enables risk stratification before starting immunotherapy and allows a close monitoring of high-risk patients during treatment.

Radiomics is a rapidly evolving research area in personalized precision medicine that aims to extract informative radiomic features from medical images and relate these features to clinical and biological endpoints. Computed tomography (CT) is routinely used for diagnosing lung cancer and assessing treatment response. CT-based quantitative radiomics approaches have been successfully applied to various tasks, such as lesion classification (Naidoo et al., 2017; Gitto et al., 2021; Xu et al., 2021), prediction of prognosis and treatment response (van Timmeren et al., 2017; Yang et al., 2019; Chetan and Gleeson, 2021), and genotype-phenotype associations (Rios Velazquez et al., 2017; Thawani et al., 2018; Zanfardino et al., 2019; Wu et al., 2021). There are very few studies focusing on the prediction of ICIP using radiomics. To the best of our knowledge, we only found two closely related ones. The first study reported a 100% accuracy of classification based on baseline chest CT images, but only two ICIP patients were enrolled (Colen et al., 2018). Mu et al. (2020) performed a radiomics analysis of PET/CT images to predict severe immune-related adverse events and achieved an area under the receiver operating characteristic curve (AUC) of 0.88 in a prospective validation cohort (Mu et al., 2020).

Predicting ICIP by conventional radiomics methods has two limitations regarding to the two steps in radiomics analysis pipeline. Radiomics requires first the segmentation of region of interest (ROI) and then the extraction of a fixed set of features from ROI. The first limitation is that it is unclear what region in pretreatment CT images should be used as ROI due to the lack of guidance for regional predilection of pneumonitis. The second limitation is that the predefined set of features may not be optimal for the final prediction task. Recent studies have demonstrated the excellent performance of deep learning models in computer-aided diagnosis (Yu et al., 2020; Zheng et al., 2020; Cheng et al., 2022b; Qian et al., 2022). Compared with hand-crafted features, deep learning models can directly learn discriminative features from images without prior segmentation of ROI and thus may provide a better prediction of ICIP.

In this study, we aim to develop a deep learning model based on clinical data and pretreatment chest CT images to predict the risk of ICIP in lung cancer patients. To this end, we collect a relatively large dataset consisting of ICIP and non-ICIP patients and propose a deep learning model in which multimodal data, two-stage transfer learning, and contrastive learning are used. Extensive experiments are conducted to assess the performance of different settings. The results demonstrate that the use of the aforementioned three strategies is effective and achieves state-of-the-art performance with an AUC of 0.918.

## 2 Materials and methods

### 2.1 Patients and data collection

**FIGURE 1:**
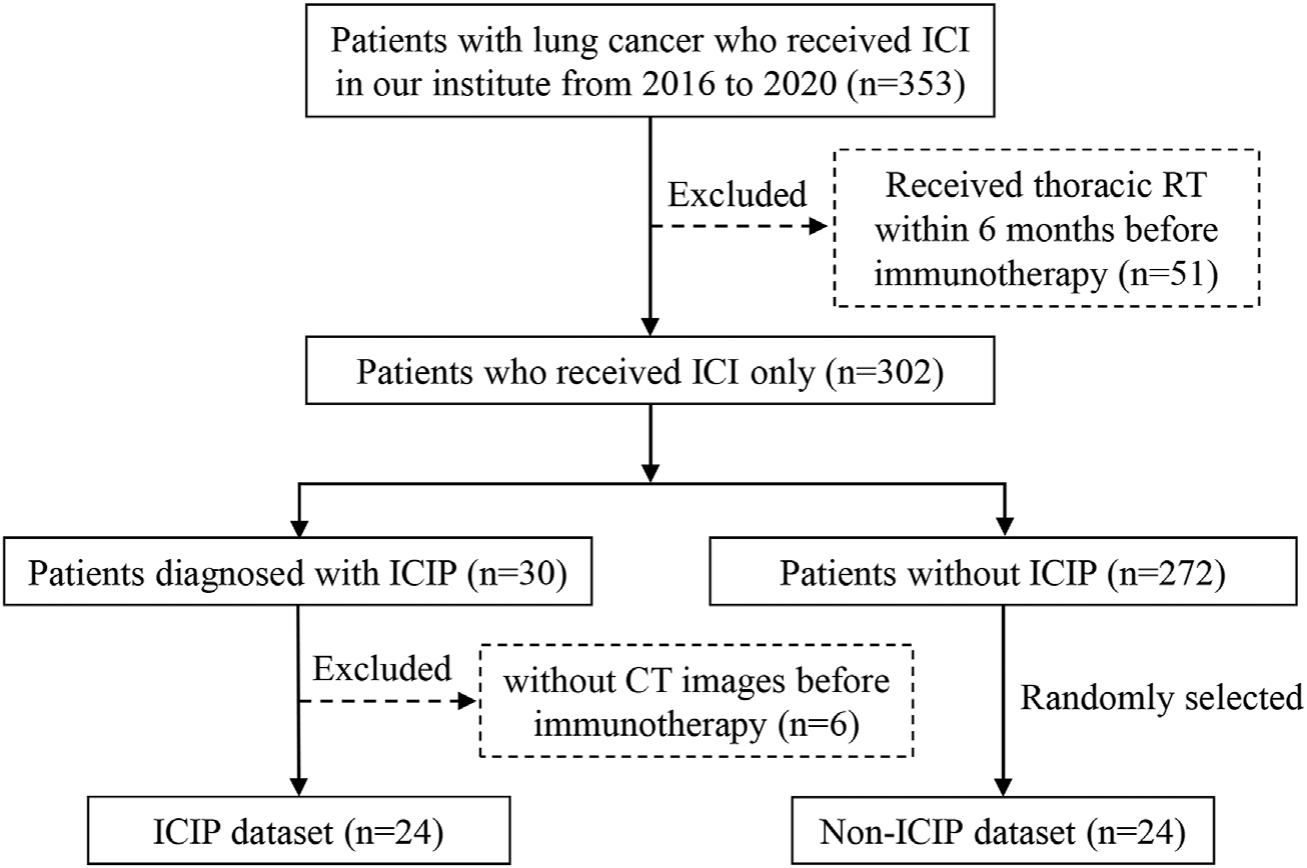
Flowchart for patient enrollment.

This study was approved by the Ethics Committee of Guangdong Provincial People’s Hospital, and the requirement for informed consent was waived. Figure 1 shows the detailed inclusion and exclusion criteria for preparing the patient cohort. A total of 353 lung cancer patients were treated with ICIs between January 2016 and December 2020 at our institute. We excluded 51 patients who received thoracic radiotherapy because radiotherapy can induce radiation pneumonitis which is difficult to be distinguished from ICIP. Among the remaining patients, 30 of them developed ICIP, resulting in an incidence of 8.50% which is comparable to the data reported in previous studies (Sears et al., 2019; Atchley et al., 2021). We used the same criteria in a previous study to define ICIP (Cheng et al., 2022a). After excluding six patients who did not have CT scans before the start of immunotherapy, we finally got 24 patients for the ICIP dataset. To match the sample size of the ICIP dataset, we randomly chose 24 patients who did not develop ICIP to construct the control dataset, i.e., the non-ICIP dataset.

The collected chest CT images are within 6 months before the start of immunotherapy, which were produced by two different scanners, Philips iCT 256 and Philips ingenuity CT. Thoracic CT scans containing the whole lung were analyzed using a multi-slice helical technique at 120 kVp, mean exposure of 205 mAs, axial resolution of 5 mm, and mean in-plane resolution of 0.8174 mm.

### 2.2 Development of the deep learning model

#### 2.2.1 Data preprocessing

For 3D CT scans, cropping, padding, and resizing techniques were used to convert the CT volume into a 192 × 192 × 224 matrix as the network input. We used a suitable window width for lung tissue from -500 to 1500 Hounsfield units (Zhang et al., 2020) to linearly rescale the pixel value to (0, 1) by the min-max method. Common data augmentation techniques including random flipping, noise, and affine transformation were used. For clinical information, as shown in Table 1, categorical variables were converted to distinct numbers so as to be input to models.

**TABLE 1:** Patient characteristics. *p* values less than 0.05 are highlighted with an asterisk.

Characteristic	ICIP dataset (*n* = 24)	Non-ICIP dataset (*n* = 24)	*p* value
**Sex**			0.022*
Female	0 (0.0%)	6 (25.0%)	
Male	24 (100.0%)	18 (75.0%)	
**Age**			0.261
Median	60	59	
Range	38–75	37–77	
**Lesion location**			0.9999
Upper left	9 (37.5%)	10 (41.7%)	
Upper right	9 (37.5%)	7 (29.2%)	
Lower left	3 (12.5%)	2 (8.3%)	
Lower right	3 (12.5%)	3 (12.5%)	
Mediastinal	0 (0.0%)	1 (4.2%)	
Middle right	0 (0.0%)	1 (4.2%)	
**Histologic type**			0.337
Adenocarcinoma	16 (66.7%)	16 (66.7%)	
Squamous cell carcinoma	7 (29.2%)	4 (16.7%)	
Adenosquamous carcinoma	0 (0.0%)	1 (4.2%)	
Small cell endocrine carcinoma	1 (4.2%)	0 (0.0%)	
Large cell endocrine carcinoma	0 (0.0%)	1 (4.2%)	
Lymphoepithelioma-like carcinoma	0 (0.0%)	2 (8.3%)	
**T stage**			0.496
T0	1 (4.2%)	1 (4.2%)	
T1	5 (20.8%)	2 (8.3%)	
T2	6 (25.0%)	11 (45.8%)	
T3	4 (16.7%)	2 (8.3%)	
T4	8 (33.3%)	8 (33.3%)	
**N stage**			0.233
N0	1 (4.2%)	2 (8.3%)	
N1	0 (0.0%)	0 (0.0%)	
N2	13 (54.2%)	7 (29.2%)	
N3	10 (41.7%)	15 (62.5%)	
**M stage**			0.461
M0	6 (25.0%)	3 (12.5%)	
M1	18 (75.0%)	21 (87.5%)	
**Surgery before immunotherapy**			0.666
Yes	4 (16.7%)	2 (8.3%)	
No	20 (83.3%)	22 (91.7%)	
**Radiotherapy before immunotherapy**			0.023*
Yes	8 (33.3%)	1 (4.2%)	
No	16 (66.7%)	23 (95.8%)	

#### 2.2.2 Network architecture

**FIGURE 2:**
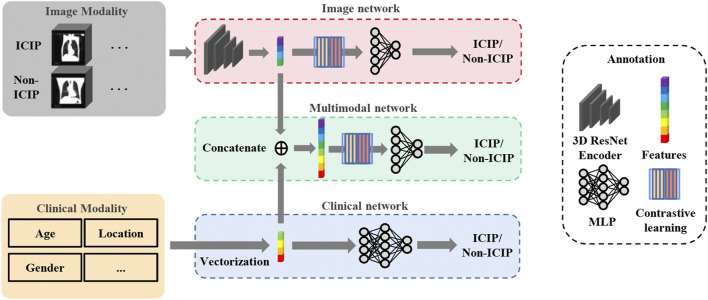
Overview of the network architecture for ICIP prediction. The top and bottom boxes show the image network and clinical network using CT images and clinical data, respectively, as input. The middle box represents the multimodal fusion network that combines image features and clinical features for ICIP prediction.

The overall network architecture is shown in Figure 2. To predict ICIP, we first built an image network and a clinical network based on pretreatment CT images and clinical data, respectively. Duo to the relatively small size of our dataset, a lightweight network, 3D ResNet18, was chosen as the backbone of the image network. Then, a multimodal fusion network was constructed by combining the nine clinical features (Table 1) and the image features learned from the image network. The clinical features and image features were fused by direct concatenation. Cross-entropy loss was used to supervise the ICIP prediction task. To enhance the prediction performance, two-stage transfer learning and contrastive learning strategies were used, which are introduced in the following sections.

#### 2.2.3 Transfer learning

**FIGURE 3:**
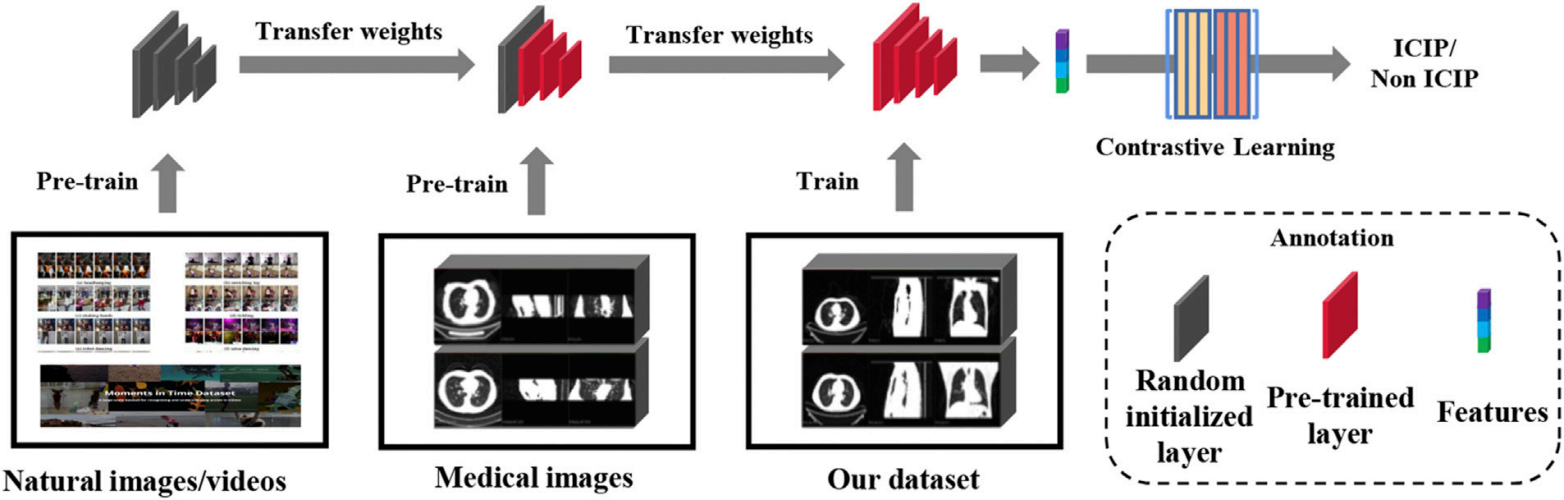
Flowchart of two-stage transfer learning.

A two-stage transfer learning strategy inspired by (Altaf et al., 2021) was used to train our image network. We first downloaded the pre-trained model which was built using two massive natural image datasets (Kay et al., 2017; Monfort et al., 2020). The pre-trained weights may not be appropriate for our ICIP prediction task due to distributional shift between natural images and medical images. Therefore, in the second stage we fine-tuned the network using a large CT image dataset associated with pneumonia (CC-CCII dataset) (Zhang et al., 2020). After the transfer of knowledge from a related task, the domain gap between the source and target tasks was significantly reduced. The two-stage transfer learning flowchart is shown in Figure 3.

#### 2.2.4 Contrastive learning

Besides transfer learning, contrastive learning was also adopted to further boost the performance of the image network. The key idea of contrastive learning is to learn an embedding space in which positive sample pairs stay close to each other while negative ones are far apart. In essence, contrastive learning allows the model to learn high-level features about the data. The contrastive learning can be broken into three basic steps: sample pair construction, encoding, and loss minimization of representations. In our study, positive sample pairs were samples from the same class while negative sample pairs were samples from different classes. We then used 3D ResNet18 to encode the images as vector representations (Figure 2). Lastly, we maximized the similarity of the two vector representations of the positive sample pair and minimized that of the negative sample pair by minimizing a contrastive loss function. We took the cosine as the similarity metric. The contrastive loss function is defined by the following equations:

$$sim\left(x_{1},x_{2}\right)=\frac{x_{1}\cdot x_{2}}{\left\|x_{1}\right\|\cdot \left\|x_{2}\right\|},$$ (1)

$$loss_{CL}=\sum_{x}sim\left(x,x_{-}\right)-\sum_{x}sim\left(x,x_{+}\right).$$ (2)

Here, sim represents the cosine similarity metric (x, x_) and (x, x_+_) respectively denote the negative sample pair and positive sample pair. The overall loss function is defined by

$$loss_{all}=\;loss_{CE}+\gamma loss_{CL},$$ (3)

where $loss_{CE}$ is the typical cross entropy loss, and γ is a hyper-parameter to control the weight of contrastive learning loss.

### 2.3 Implementation details

The proposed method was implemented using PyTorch on a workstation equipped with four NVIDIA RTX A6000 GPUs (48 GB memory each). In all comparative studies, we employed ResNet18 (He et al., 2016) as the backbone. Adam optimizer with the learning rate of 1e-4, 1e-4, and 5e-5 was employed to train the baseline model (without transfer learning), one-stage transfer learning model, and two-stage transfer learning model, respectively. The batch size and γ were set to 6 and 0.02, respectively, which were selected by grid search. Specifically, a finite number of values were tried, and the one with the best performance was selected. In all experiments, five-fold cross-validation was used, and the average performance was reported.

### 2.4 Statistical analysis

To compare the data distribution between the ICIP and non-ICIP datasets, Fisher’s exact test was used for categorical variables, and Mann-Whitney U test was used for continuous variables. Two-tailed tests are used to determine significance at the 5% level. All statistical analyses were conducted using Statistical Product and Service Solutions (IBM SPSS, version 20.0).

To evaluate the classification performance, several typical metrics were used, including accuracy, sensitivity, specificity, precision, and F1-score. We considered ICIP as the positive class and non-ICIP as the negative class, so true positive (TP), false positive (FP), true negative (TN), and false negative (FN) can be accordingly defined. After getting the numbers of TP, FP, TN, and FN, the abovementioned performance metrics can be calculated using Eqs. 4–8. Since we used five-fold cross-validation, the average of these metrics were reported. We also used the area under the receiver operating characteristic (ROC) curve to evaluate model performance. Since every patient was tested for and only for once in five-fold cross-validation, we gathered the results across all the five folds, then plotted ROC curves, and calculated AUCs.

$$Accuracy=\frac{TP+TN}{TP+FP+TN+FN}$$ (4)

$$Sensitivity=\frac{TP}{TP+FN}$$ (5)

$$Specificity=\frac{TN}{TN+FP}$$ (6)

$$Precision=\frac{TP}{TP+FP}$$ (7)

$$F1-score=\frac{2Precision*Specificity}{Precision+Specificity}$$ (8)

## 3 Results

### 3.1 Patient characteristics

Among the 48 patients, there were 42 men and 6 women with an overall mean age of 58.00 years ± 9.75 (standard deviation). We collected nine clinical characteristics for the 48 patients. Table 1 shows these characteristics separately for the ICIP and non-ICIP datasets. Among the nine characteristics, there were significant differences between the two datasets for sex (Fishers’ exact test *p* value = 0.022) and radiotherapy before immunotherapy (Fishers’ exact test *p* value = 0.023), whereas no significant differences were observed for the remaining characteristics.

### 3.2 Performance of deep learning model to predict Immune checkpoint inhibitors-related pneumonitis

To explore and validate the effectiveness of the key components used in our method, we conducted extensive experiments. The comparison of quantitative performance is presented in Table 2. The details of different methods are provided as follows:

• Cli denotes the clinical network
• Im denotes the image network without using transfer learning and contrastive learning
• CI denotes the multimodal network built on both clinical data and CT images
• Im-1T denotes the image network with one-stage transfer learning
• Im-2T denotes the image network with two-stage transfer learning
• Im-2T-C denotes the image network with two-stage transfer learning and contrastive learning
• CI-2T denotes the multimodal network with two-stage transfer learning
• CI-2T-C denotes the multimodal network with two-stage transfer learning and contrastive learning

**TABLE 2:** Quantitative analysis of key components in our method. The best results are highlighted in bold.

Method	AUC	Accuracy	Sensitivity	Specificity	Precision	F1-score
Cli	0.701	0.730 ± 0.045	0.660 ± 0.134	0.800 ± 0.200	0.817 ± 0.171	0.723 ± 0.041
Im	0.753	0.725 ± 0.075	0.740 ± 0.195	0.710 ± 0.175	0.728 ± 0.079	0.717 ± 0.081
CI	0.797	0.815 ± 0.078	0.790 ± 0.143	0.840 ± 0.089	0.837 ± 0.096	0.814 ± 0.079
Im-1T	0.821	0.830 ± 0.120	0.700 ± 0.200	0.960 ± 0.089	0.837 ± 0.096	0.824 ± 0.125
Im-2T	0.854	0.855 ± 0.087	0.800 ± 0.200	0.910 ± 0.125	0.920 ± 0.110	0.851 ± 0.090
Im-2T-C	0.901	0.920 ± 0.084	0.960 ± 0.089	0.880 ± 0.179	0.910 ± 0.131	0.918 ± 0.087
CI-2T	0.865	0.880 ± 0.130	0.920 ± 0.110	0.840 ± 0.167	0.860 ± 0.142	0.879 ± 0.131
CI-2T-C	0.918	0.920 ± 0.084	0.920 ± 0.110	0.920 ± 0.179	0.943 ± 0.128	0.918 ± 0.087

#### 3.2.1 Effectiveness of multimodal data fusion

**FIGURE 4:**
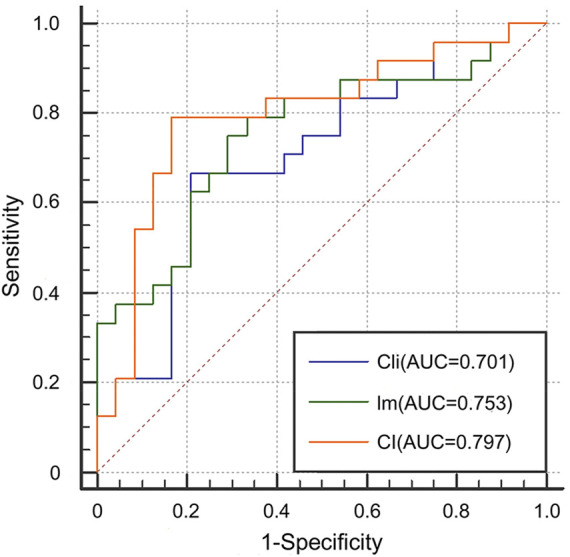
ROC curves for the Cli, Im, and CI networks to show the effectiveness of multimodal data fusion.

We first evaluated the effectiveness of combining images and clinical data to predict ICIP. To this end, we compared the classification performance of the multimodal network with that of the image network and the clinical network (CI vs. Cli and Im, Table 2). As shown in Table 2, the image network achieved an AUC of 0.753 (Im, Table 2), which was superior to the clinical network that yielded an AUC of 0.701 (Cli, Table 2). By utilizing both images and clinical data, the classification performance was significantly improved up to 0.797 (CI, Table 2). Other metrics in Table 2 also indicates that multimodal data fusion is beneficial to ICIP prediction. ROC curves are shown in Figure 4.

#### 3.2.2 Effectiveness of two-stage transfer learning

**FIGURE 5:**
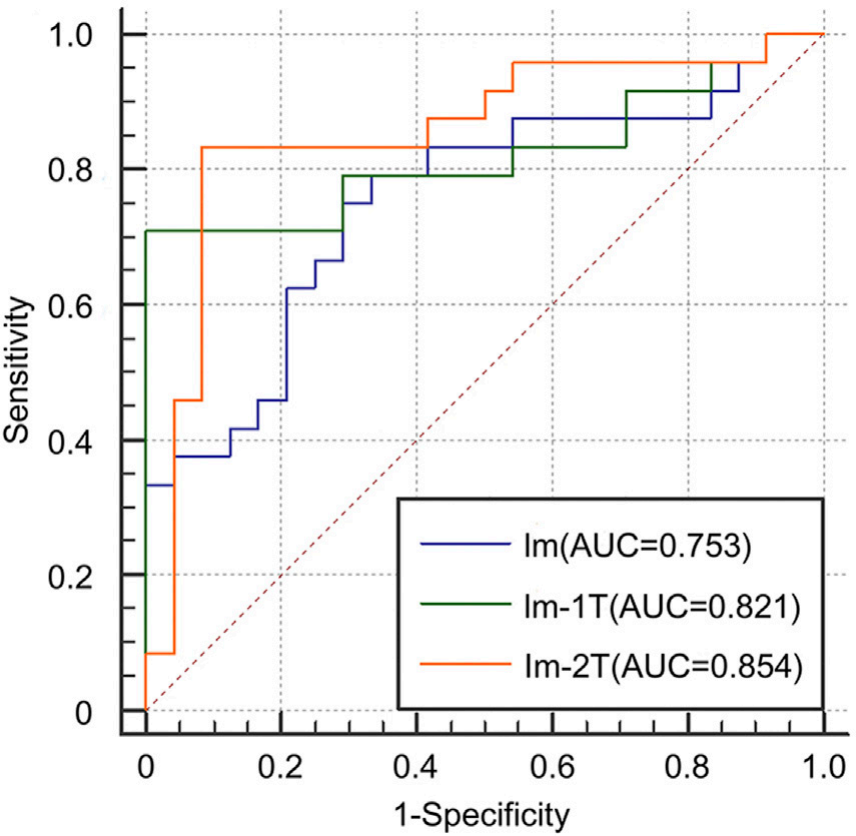
ROC curves for the Im, Im-1T, and Im-2T networks to show the effectiveness of two-stage transfer learning.

We next evaluated the effectiveness of the proposed two-stage transfer learning strategy. To this end, we first used the image network without transfer learning as the baseline and then gradually incorporated one-stage and two-stage transfer learning. Compared with the baseline model trained from scratch, one-stage transfer learning brought large performance gain from 0.753 to 0.821 in term of AUC (Im vs. Im-1T, Table 2). Moreover, the use of the two-stage transfer learning further lifted the prediction performance. The AUC, accuracy, sensitivity, and specificity were 0.854, 0.855, 0.800, and 0.910, respectively (Im-2T, Table 2). The increasingly better performance from Im, Im-1T to Im-2T suggests that using a pre-trained network and fine-tuning on a large related dataset are essential to obtain good performance. ROC curves are shown in Figure 5.

#### 3.2.3 Effectiveness of contrastive learning

**FIGURE 6:**
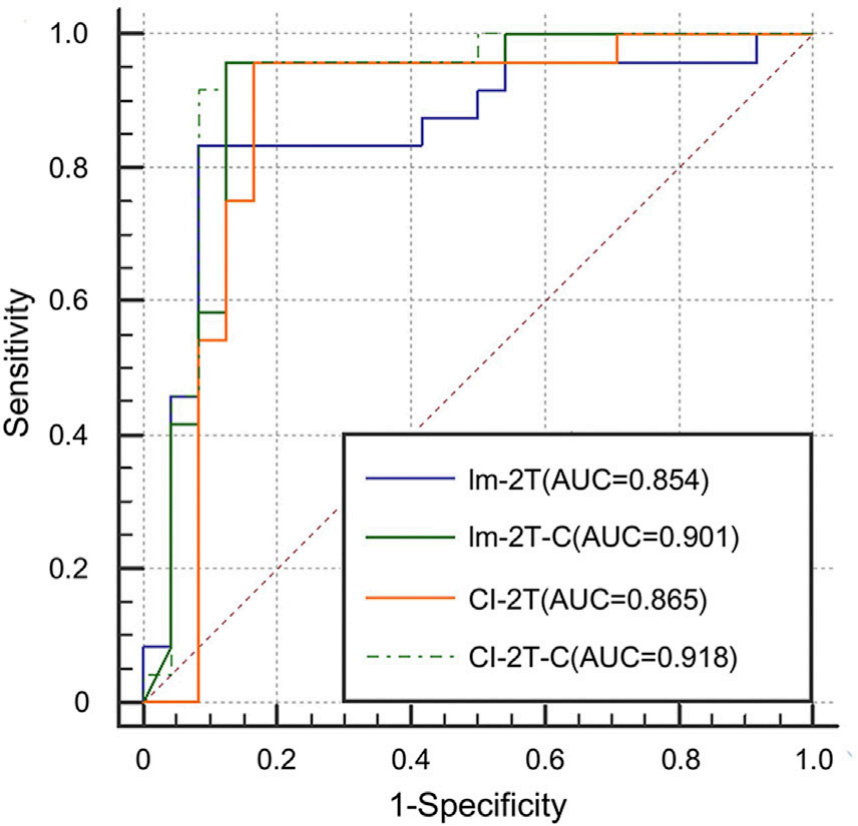
ROC curves for the Im-2T, Im-2T-C, CI-2T, and CI-2T-C networks to show the effectiveness of contrastive learning.

Finally, we evaluated the effectiveness of the contrastive learning strategy in the image network and the multimodal network. The image network with two-stage transfer learning but without contrastive learning achieved AUC of 0.854, accuracy of 0.855, sensitivity of 0.800, and specificity of 0.910 (Im-2T, Table 2). Adding contrastive learning gave a boost in performance. The resulting AUC, accuracy, sensitivity, and specificity were 0.901, 0.920, 0.960, and 0.880, respectively (Im-2T-C, Table 2). Similarly, performance gain was also observed when incorporating contrastive learning into the multimodal network. The multimodal network with two-stage transfer learning yielded AUC of 0.865, accuracy of 0.880, sensitivity of 0.920, and specificity of 0.840 (CI-2T, Table 2). The use of contrastive learning increased the performance by a large margin. The resulting AUC, accuracy, sensitivity, and specificity were 0.918, 0.920, 0.920, and 0.920, respectively (CI-2T-C, Table 2). ROC curves are shown in Figure 6.

### 3.3 Visualization

**FIGURE 7:**
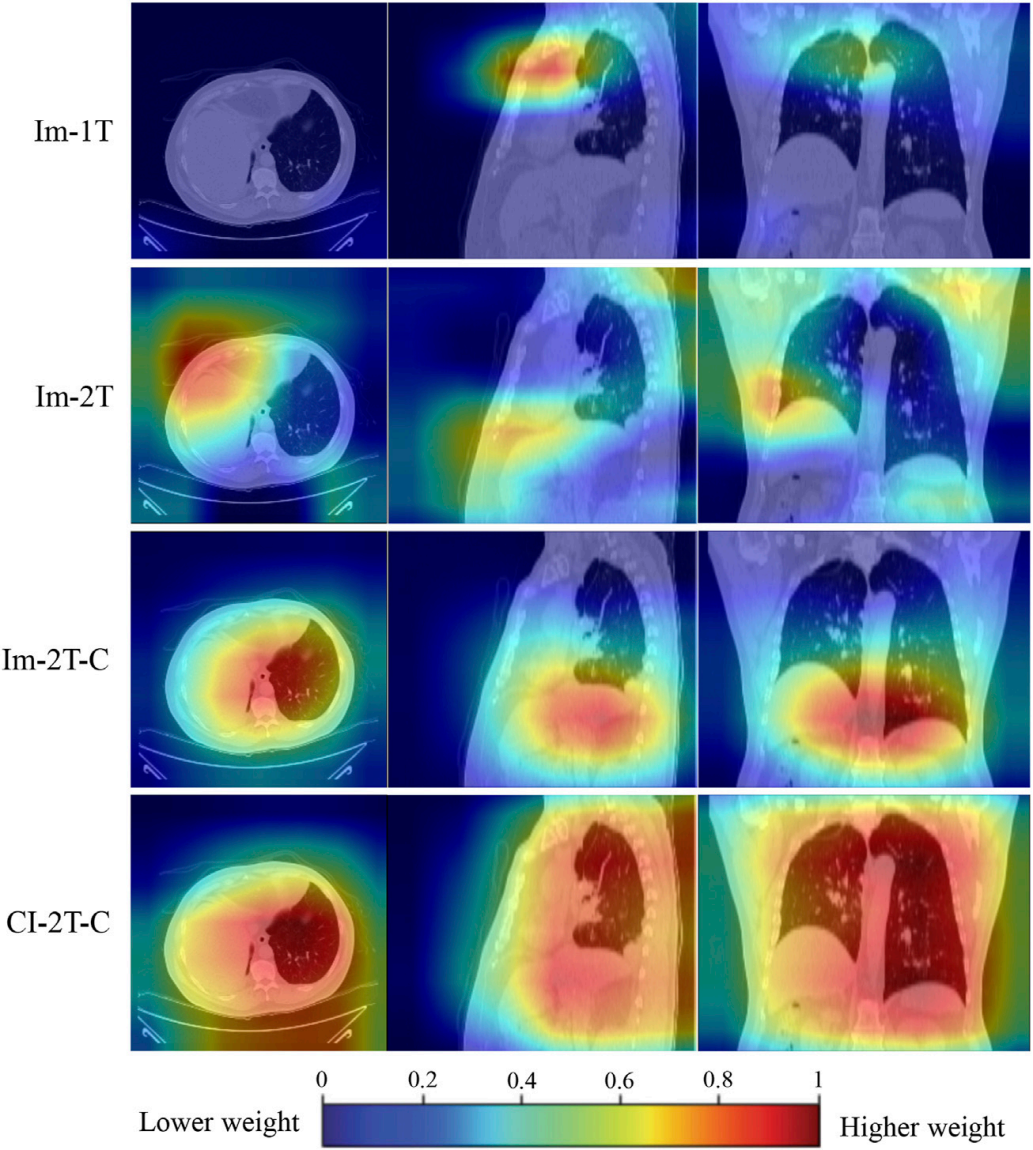
Activation maps for a non-ICIP sample to show the most “important” regions that different methods consider. The red color represents a higher weight (i.e., more attention is paid to this region).

To gain an understanding of why the performance was improved when the key components such as two-stage transfer learning were introduced to the model, the class activation maps were generated by the gradient of the deep learning to highlight the important regions within the input image (Gotkowski et al., 2021). As shown in Figure 7, the primary attention of the one-stage transfer learning model (Im-1T, first row) was not focused on the lung area. However, the use of two-stage transfer learning and especially contrastive learning brought more attention to the lung area (Im-2T and Im-2T-C, second and third rows). Interestingly, fusing clinical and image features made the network concentrate on the whole lung (CI-2T-C, fourth row), indicating that the whole lung is crucial and informative for ICIP prediction. This makes sense as ICIP can occur anywhere in lung.

## 4 Discussion

As ICI treatment is becoming more frequently used in lung cancer patients, an increasing number of irAEs (i.e., ICIP) are being reported. ICIP is potentially fatal. Thus, early prediction of ICIP is crucial for improving treatment outcomes. However, based on clinical factors or pretreatment CT images, it is very challenging for doctors to predict whether ICIP will occur prior to immunotherapy. Therefore, there is a critical need for an accurate and automated approach to assist doctors in identifying patients at risk for ICIP before immunotherapy, which allows personalized treatment options and reduces the number of deaths due to severe ICIP. In this study, we developed the first deep learning model for predicting ICIP using clinical information and pretreatment baseline chest CT images. In addition to the use of multimodal data, we also introduced two-stage transfer learning and contrastive learning in our model development. We evaluated our method using five-fold cross-validation on 24 ICIP patients and 24 non-ICIP patients. The results demonstrated that the deep learning model accurately differentiated between ICIP and non-ICIP patients, with an AUC of 0.918.

Few prior studies have demonstrated the utility of radiomics to predict irAEs. Colen et al. (2018) presented the first reported work exploring the potential of CT-based radiomics to predict patients at risk for developing ICIP and reported an AUC of 1 (Colen et al., 2018). Although the performance was extremely high, this study only included 2 ICIP cases and suffered from severe class imbalance problem. By contrast, our study used a balanced dataset consisting of 24 ICIP cases and 24 non-ICIP cases. Mu et al. proposed a PET/CT based radiomics approach to predict severe irAEs in patients with NSCLC (Mu et al., 2020). A total of 30 cases with severe irAEs and 164 control cases were curated in the patient cohorts. The radiomics approach yielded an AUC of 0.88 in the prospective validation cohort. However, this work is based on PET/CT which is not widely available in hospitals and thus has limited utility. In contrast to the traditional radiomics methods that extracted a fixed set of image features, our study proposed a deep learning model that can directly learn discriminative features from CT images and demonstrated a better performance with an AUC of 0.918.

The superiority of our method can be attributed to the use of multimodal data fusion, two-stage transfer learning, and contrastive learning in our deep learning model. The effectiveness of these key components was validated by extensive ablation studies. The multimodal data fusion model outperformed the models built on either clinical data or CT images by a large margin. This suggests that the two kinds of data harbor complementary information. Thus, the ICIP prediction task can greatly benefit from this fusion approach. Training deep learning models requires a large dataset. However, in medical applications, oftentimes, only a small dataset is available due to low incidence of disease or expensive cost of data collection. Our results confirm that transfer learning is helpful in this case. Simply using a pre-trained model learned on a large unrelated dataset (one-stage transfer learning) or subsequently retraining the model on a large related dataset (two-stage transfer learning) can improve the performance of ICIP prediction markedly. Moreover, contrastive learning can further enhance the feature representation ability by contrasting similar (positive) and dissimilar (negative) samples.

This study has several limitations. First, although our method was rigorously validated by five-fold cross-validation, the data used in this study was collected from a single institution, future efforts will concentrate on validating the findings in a larger multi-institutional cohort. Second, to maintain a balance of sample size between classes, we randomly selected a portion of patients without ICIP to match the sample size of the ICIP dataset. There might be an issue with this strategy as it does not reflect a real-world class distribution. Third, duo to the retrospective nature of this study, it may be prone to biases from missing data and reliance on available medical documentation for review. Prospective studies are needed in the future.

In conclusion, patients who will develop ICIP have subtle changes at their pretreatment baseline CT scans that could not be identified by the naked eye but could be detected by quantitative analysis. Our study presents the first deep learning model based on clinical data and CT images to predict patients at risk for developing ICIP. This model can accurately predict ICIP patients with an AUC of 0.918, which enables efficient risk stratification, close monitoring, and prompt management of ICIP. This will potentially improve ICI treatment outcomes in patients with lung cancer.

## Data Availability

The original contributions presented in the study are included in the article/supplementary material, further inquiries can be directed to the corresponding authors.
